# Supplementary material for: Doublet decoding of tRNASer3 demonstrates plasticity of ribosomal decoding center
Source: Nat Commun. 2025 Jun 26;16:5402. doi: 10.1038/s41467-025-61016-5 (PMC12202706; doi:10.1038/s41467-025-61016-5)
Supplement: Supplementary file 1 — Supplementary Information [file 41467_2025_61016_MOESM1_ESM.pdf]

Supplementary material for

Doublet decoding of tRNA^{Ser3} demonstrates plasticity of ribosomal decoding center

Shruthi Krishnaswamy[1,2#], Shirin Akbar[1#], Daniel S. D. Larsson[1#], Yang Chen[1,3] & Maria Selmer[1]*

[1.] Department of Cell and Molecular Biology, Uppsala University, BMC, P.O. Box 596, SE-75124 Uppsala, Sweden

[2.] Present address: Department of Integrated Structural Biology, Institut de Génétique et de Biologie Moléculaire et Cellulaire (IGBMC), 67400, Illkirch, France

[3.] Present address: MAX IV Laboratory, Lund University, P.O. Box 118, SE-221 00 Lund, Sweden

[#] These authors contributed equally

*To whom correspondence should be addressed: maria.selmer@icm.uu.se, tel. +46 18 4714177

Content:

Supplementary Tables 1-3

Supplementary Figures 1-11

Supplementary References

Supplementary Table 1. RNA oligos. Anticodons and A-site codons in bold.

ASL^{Ala1}	CCUGCUU**UGC**ACGCAGG
ASL^{Ser3}	CUCCCCU**GCU**AAGGGAG
ASL^{Ser3} U36C	CUCCCCU**GCC**AAGGGAG
mRNA-AGC	GGCAAGGAGGUAAAAAUG**AGC**AAA
mRNA-GCA	GGCAAGGAGGUAAAAAUG**GCA**AAA

Supplementary Table 2. DNA oligos.

ser3_exp_f1 (EcoRI)	GGAATTCTGTGGTGAGGTGGCCGAGAGGCTGAAGGCGCTCCCCT GCTAAGGGAGTATGCGGTCAAAAGCT
ser3_exp_b1 (PstI)	AACTGCAGGCAAATGGCGGTGAGGCGGGGATTCGAACCCCGGAT GCAGCTTTTGACCGCATACTCCC

Supplementary Table 3. Cryo-EM data collection, refinement and validation statistics

	#1 Cognate (EMDB-51758) (PDB 9H0L)	#2 Doublet decoding (EMDB-51679) (PDB 9GXX)
**Data collection and processing**		
Magnification	190,000	190,000
Voltage (kV)	200	200
Electron exposure (e–/Å^2)	28.5	28.3
Defocus range (μm)	-0.7 to -1.3	-0.7 to -1.3
Pixel size (Å)	0.7463	0.7463
Symmetry imposed	C1	C1
Initial particle images (no.)	751,778	1,051,022
Final particle images (no.)	167,987	196,921
Map resolution (Å)	2.49	2.61
FSC threshold	0.143	0.143
Map resolution range (Å)	1.86–43.6	1.91–41.8
**Refinement**		
Initial models used (PDB codes)	7K00, 8CGK, 8CGJ, 8CF1	7K00, 8CGK, 8CGJ, 8CF1
Model resolution (Å)	2.65	2.78
FSC threshold	0.5	0.5
Map sharpening *B* factor (Å^2)	-64.9	-67.2
Model composition		
Non-hydrogen atoms	144,192	144,033
Protein residues	5,587	5,587
RNA residues	4,657	4,648
Waters	0	0
Mg^{2+}	314	288
B factors (Å^2)	114	114
Protein	122	121
RNA	111	111
R.m.s. deviations		
Bond lengths (Å)	0.009	0.009
Bond angles (°)	1.70	1.52
Validation		
MolProbity score	1.18	1.21
Clashscore	2.35	2.91
Poor rotamers (%)	0.87	0.72
Ramachandran plot		
Favored (%)	97.06	97.32
Allowed (%)	2.90	2.66
Disallowed (%)	0.04	0.02
Rama-Z score	-1.13	-1.15

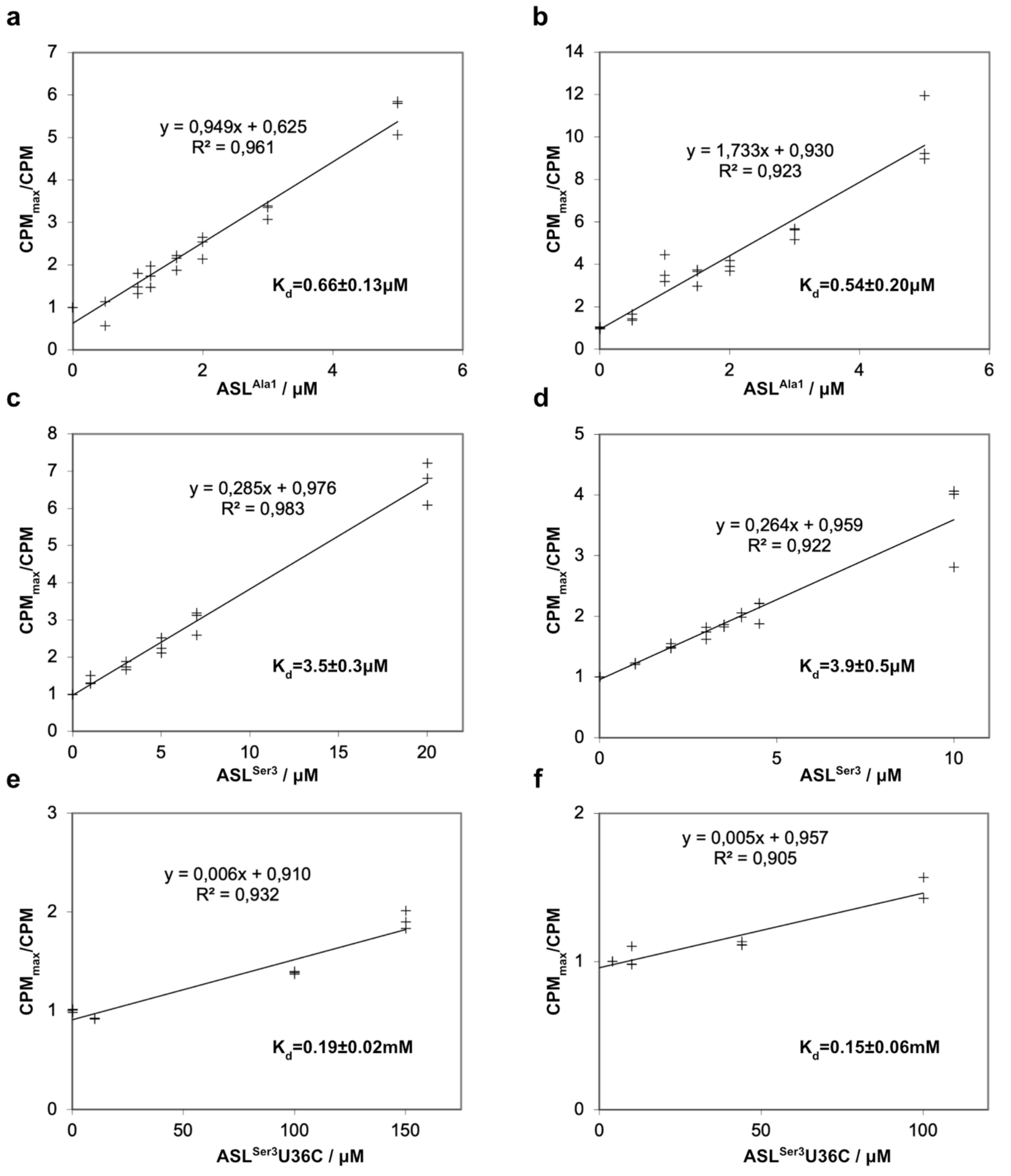

Supplementary Figure 1. Nitrocellulose filter binding plots of ASL^{Ala1} (**a, b**), ASL^{Ser3} (**c, d**) and ASL^{Ser3}U36C (**e, f**) to the A-site GCA codon of the *E. coli* 70S ribosome. For each concentration n=3 independent measurements. For each ASL, n=2 technical replicates. The X axis shows the concentration of cold competitor ASL and the Y axis the ratio of the background-subtracted CPM values at zero concentration (no competitor) and at the indicated concentration. From this plot, K_d is derived as the ratio between the intercept and the slope of the fitted line. The sum of percentage standard deviation of the intercept and the slope is summed and applied to the K_d.

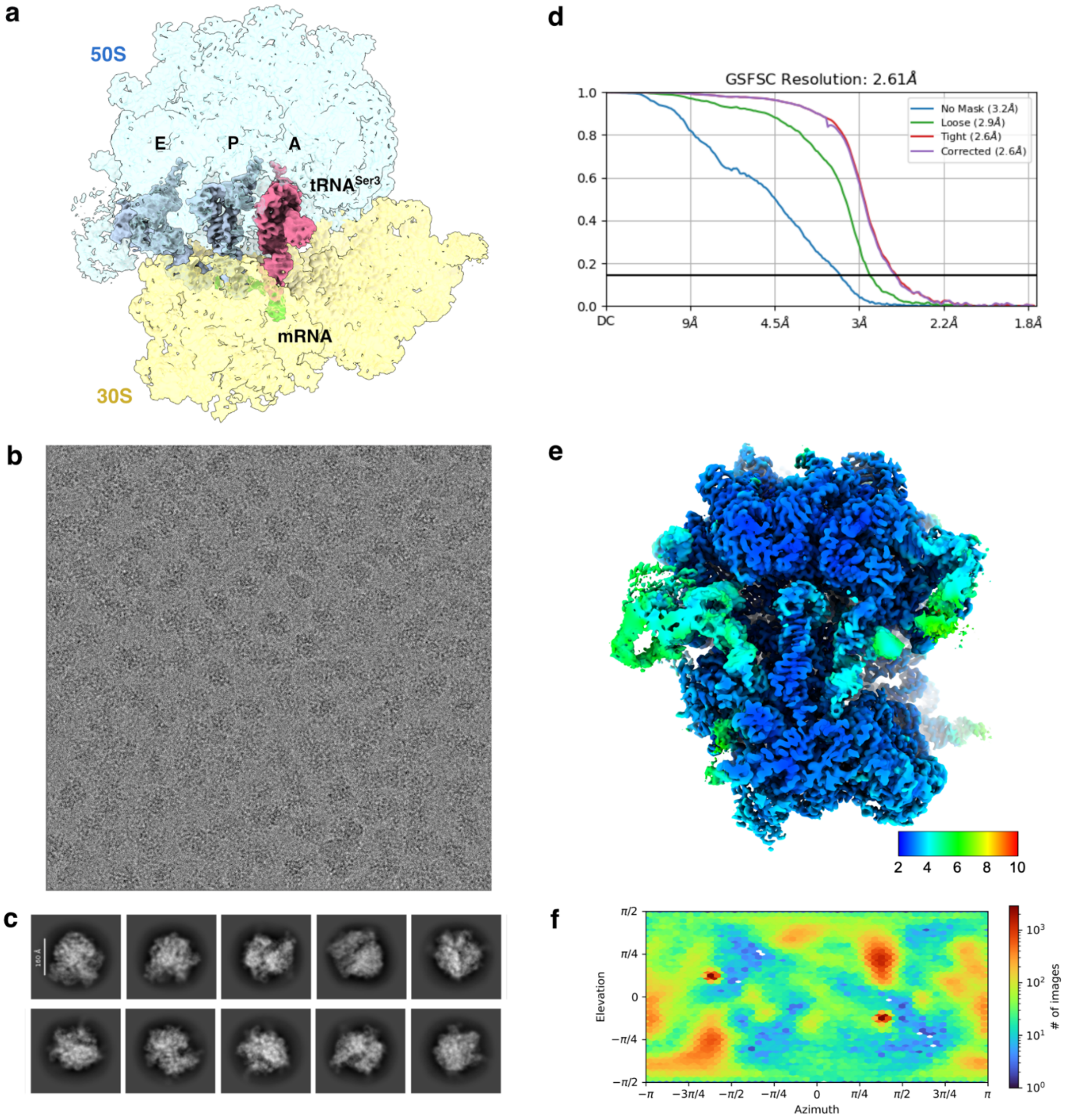

Supplementary Figure 2. Cryo-EM reconstruction of a doublet-decoding 70S ribosomal complex with mRNA-GCA and tRNA^{Ser3}. (**a**) 3D reconstruction shown with tRNA^{Ser3} in dark pink, tRNA^{fMet} and E-site tRNA in blue-gray, mRNA in green, 30S subunit in yellow and 50S subunit in blue. (**b**) Representative micrograph. (**c**) 2D class averages. (**d**) FSC plot. (**e**) Local resolution (in Å) in a slice at the tRNA binding sites. (**f**) Angular distribution.

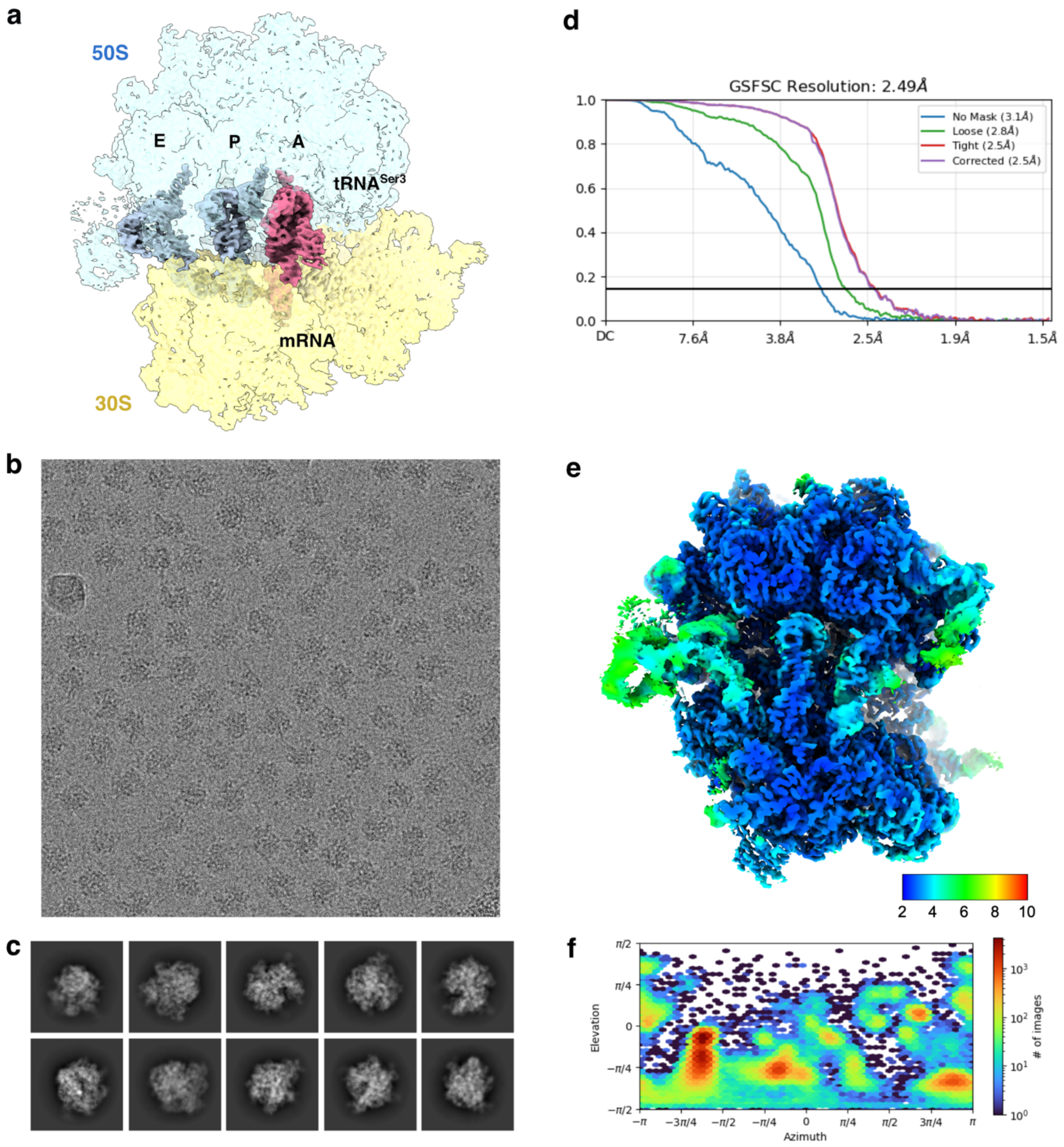

Supplementary Figure 3. Cryo-EM reconstruction of cognate 70S ribosomal complex with mRNA-AGC and $tRNA^{Ser3}$. (**a**) 3D reconstruction shown with $tRNA^{Ser3}$ in dark pink, $tRNA^{fMet}$ and E-site tRNA in blue-gray, mRNA in green, 30S subunit in yellow and 50S subunit in blue. (**b**) Representative micrograph (**c**) 2D class averages. (**d**) FSC plot. (**e**) Local resolution (in Å) in a slice at the tRNA binding sites. (**f**) Angular distribution.

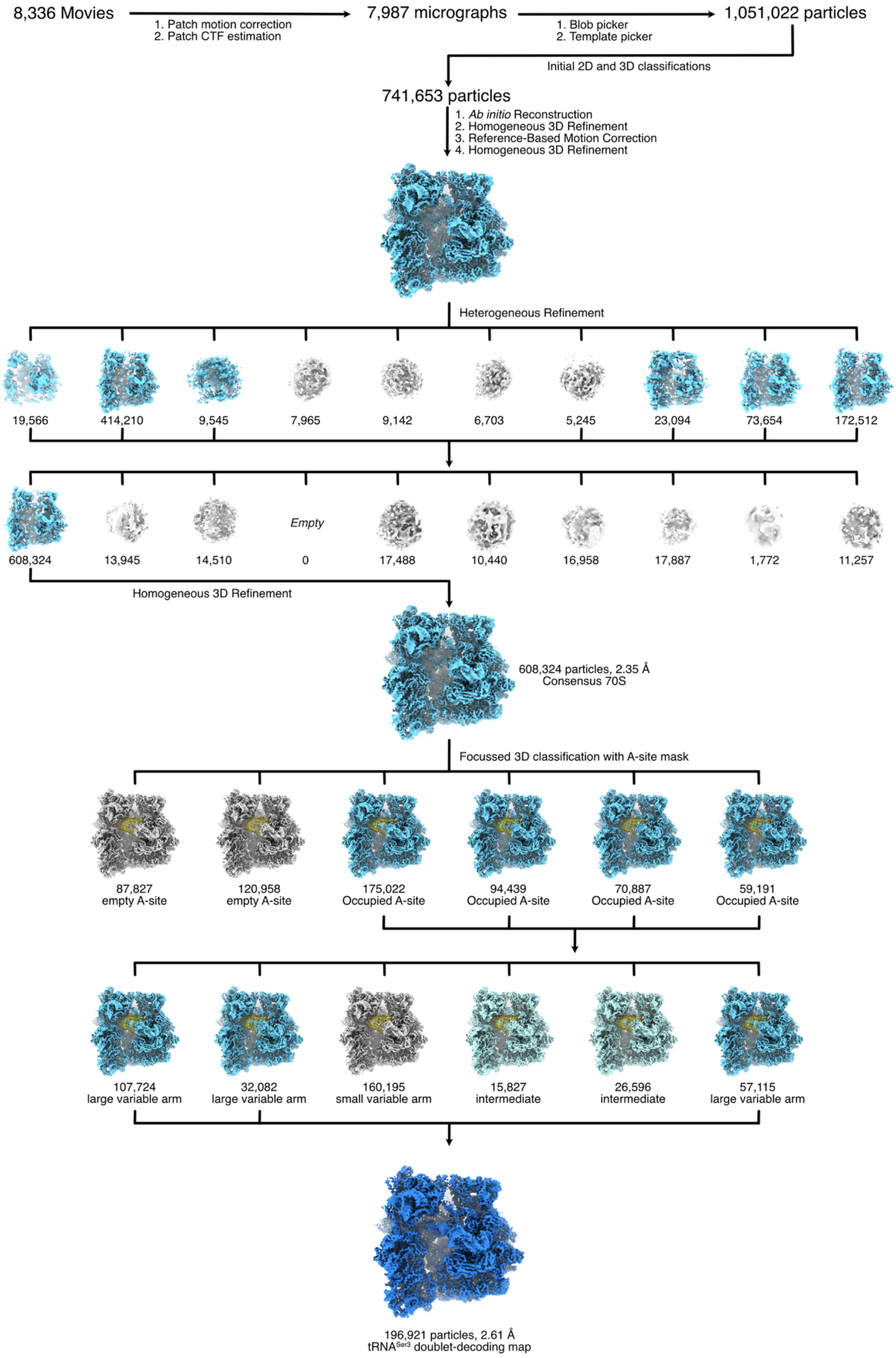

Supplementary Figure 4. Cryo-EM data processing workflow for the doublet-decoding structure. Focused mask region for the A-site classification is shown in red outline.

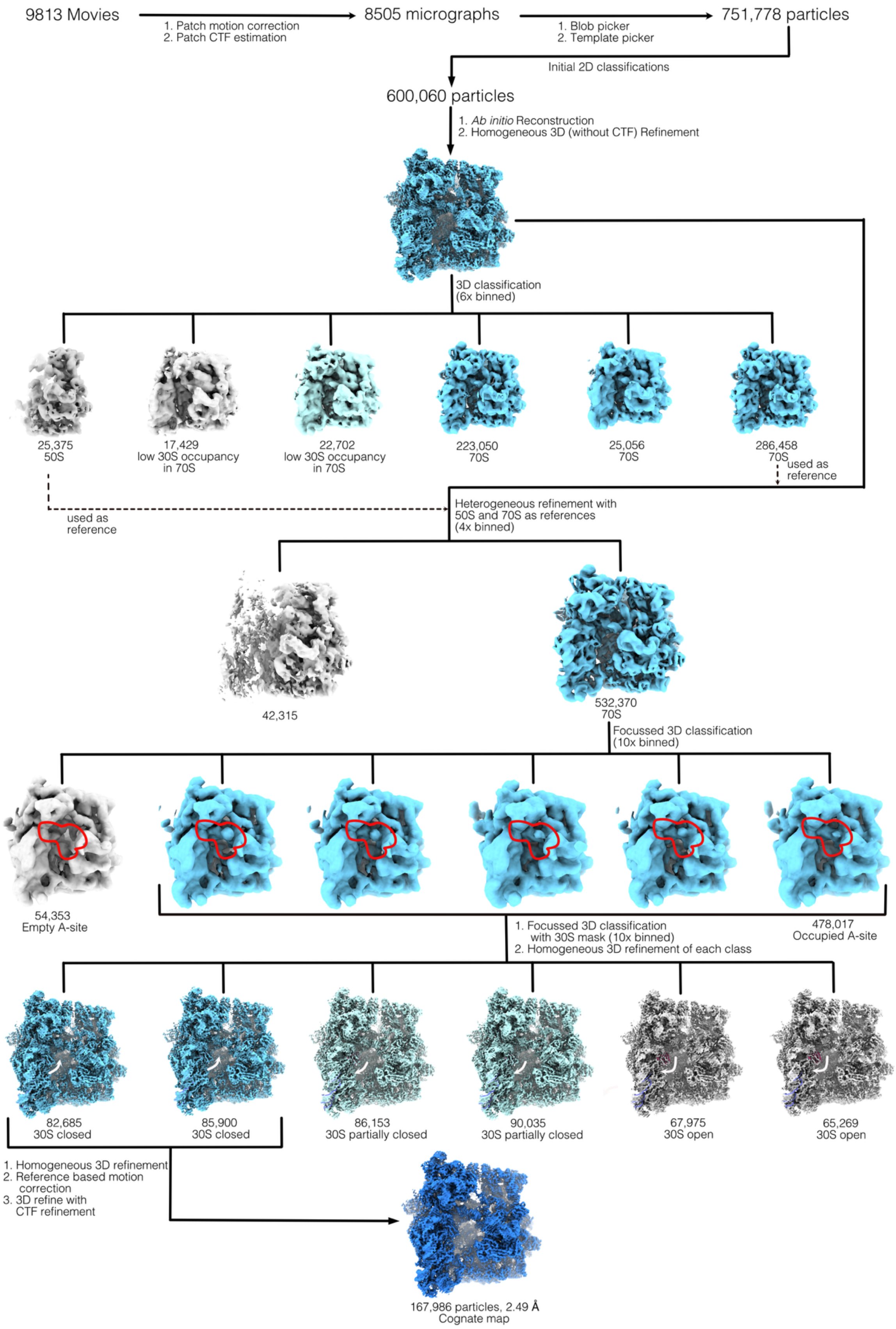

Supplementary Figure 5. Cryo-EM data processing workflow for the cognate structure. Focused mask region for the A-site classification is shown in red outline. 30S conformation shown with inward or outward facing white arrows for closed or open conformation respectively.

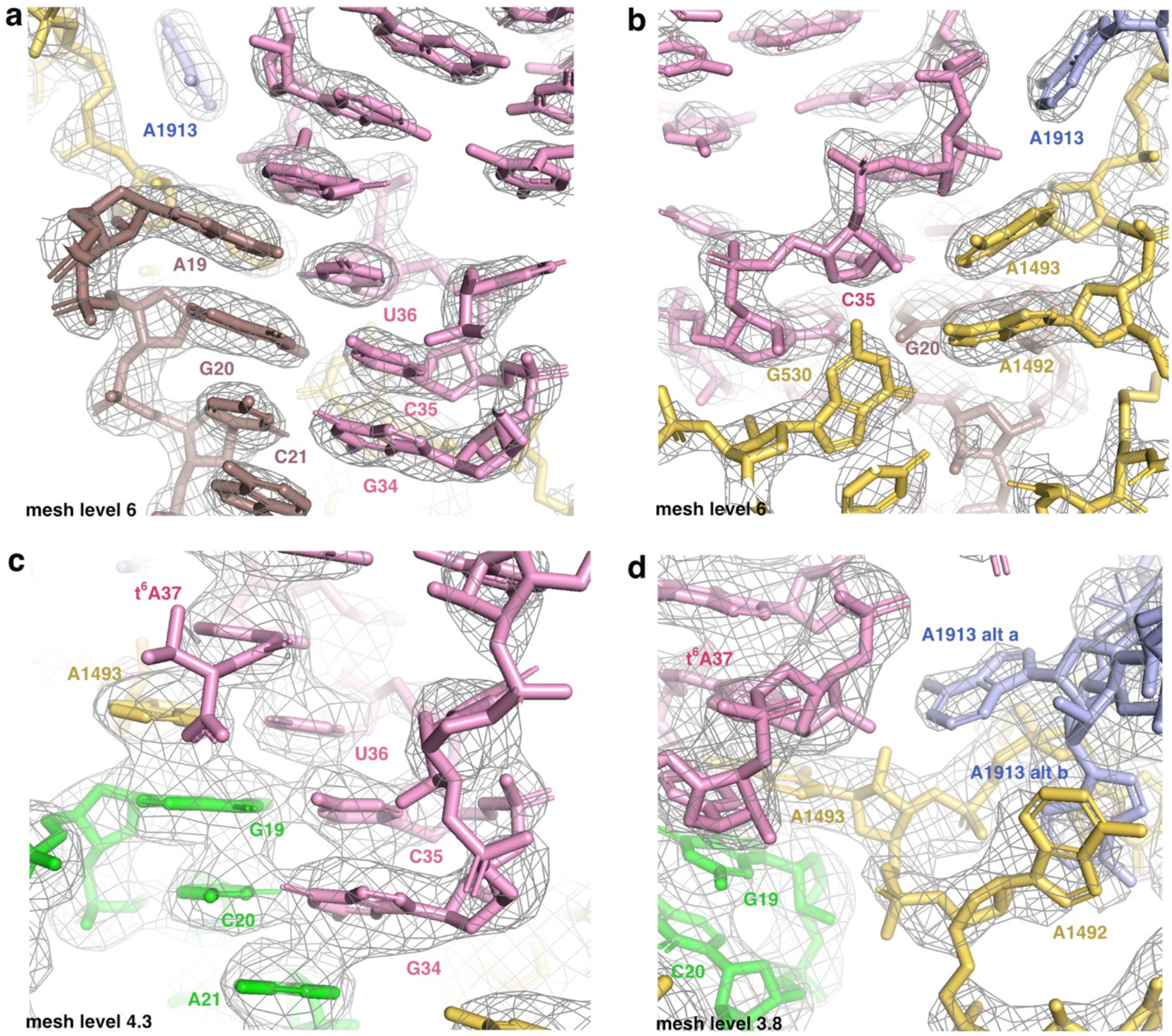

Supplementary Figure 6. Cryo-EM map density shown in mesh with fitted coordinates for the cognate (**a, b**) and doublet-decoding (**c, d**) structures. 16S rRNA is shown in yellow, 23S rRNA in light blue, $tRNA^{Ser3}$ in pink. mRNA-AGC is shown in brown (cognate structure) and mRNA-GCA in green (doublet-decoding structure). PyMOL mesh level for individual maps are indicated (bottom left). To visualize the two alternative conformations of A1913, the map threshold is lower in (d) than in (c). The alt a conformation of A1913 is equivalent to the cognate structure (b), where A1913 forms a hydrogen bond to the 2’O of t^6A37.

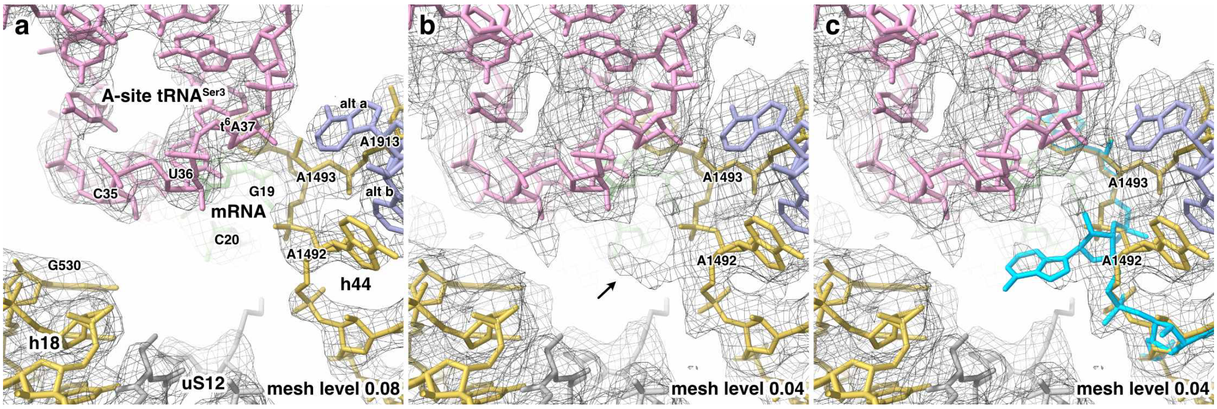

Supplementary Figure 7. Conformational dynamics of A1492 in the doublet-decoding structure. (**a**) The map supports the model where A1492 stacks with A1913 (alt b) and A1493 forms a Hoogsteen base pair to anticodon base U36 of $tRNA^{Ser3}$. (**b**) At lower mesh level, there is weak density (black arrow) suggesting that A1492 in a minor conformation extends towards the tRNA anticodon. (**c**) Map as in (b) with geometrically sound alternative conformation of A1492-A1493 (shown in cyan). $tRNA^{Ser3}$ is shown in pink, 16S rRNA in yellow, 23S rRNA in light blue, mRNA in green and r-protein S12 in gray.

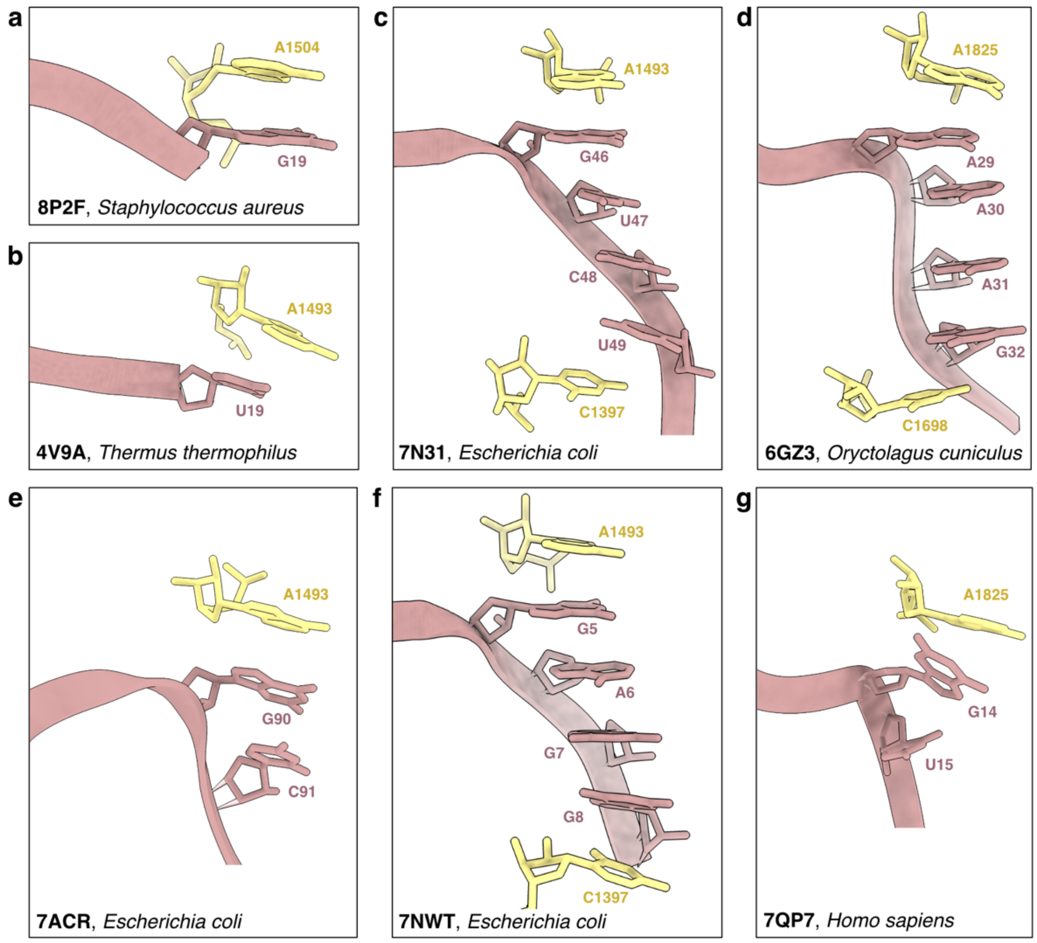

Supplementary Figure 8. Identified structures (PDB code indicated) where A1493 or its equivalent is stacked with the first base of the A-site codon. 16S/18S rRNA is shown in yellow and mRNA in brown. **(a)** 70S ribosome with EF-G and fusidic acid cyclopentane (cryo-EM) [1]. **(b)** 70S ribosome with tetracycline. (X-ray crystallography) [2]. **(c)** Elongating 70S ribosome complex in a post-translocation (POST) conformation (cryo-EM) [3]. **(d)** tRNA translocation by the eukaryotic 80S ribosome and the impact of GTP hydrolysis. Translocation-intermediate-POST-1 (TI-POST-1) (cryo-EM) [4]. **(e)** Post-translocated trans-translation complex on stalled ribosome (cryo-EM) [5]. **(f)** Initiated 70S ribosome in complex with 2A protein from encephalomyocarditis virus (cryo-EM) [6]. **(g)** Structure of the human 48S initiation complex in closed state (cryo-EM) [7].

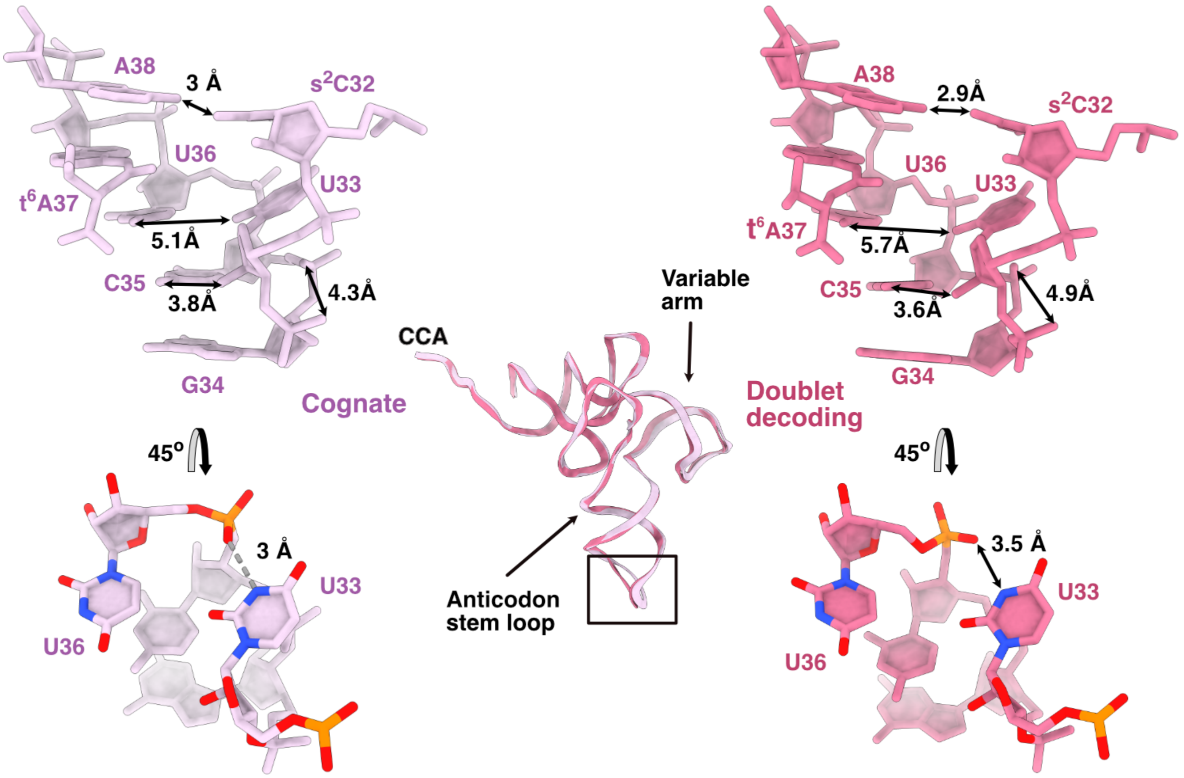

Supplementary Figure 9. Comparison of the anticodon loop of tRNA^{Ser3} at the A-site of the cognate (pale pink) and doublet-decoding (dark pink) structures. Center: overlay between the two conformations of tRNA^{Ser3}with a box around the anticodon loop region shown in the other panels. Distances that differ between the two structures are shown with double-headed arrows. Lower panel shows that widening of the anticodon loop in the doublet-decoding complex results in breaking of a hydrogen bond between the U33 base and the U36 backbone phosphate.

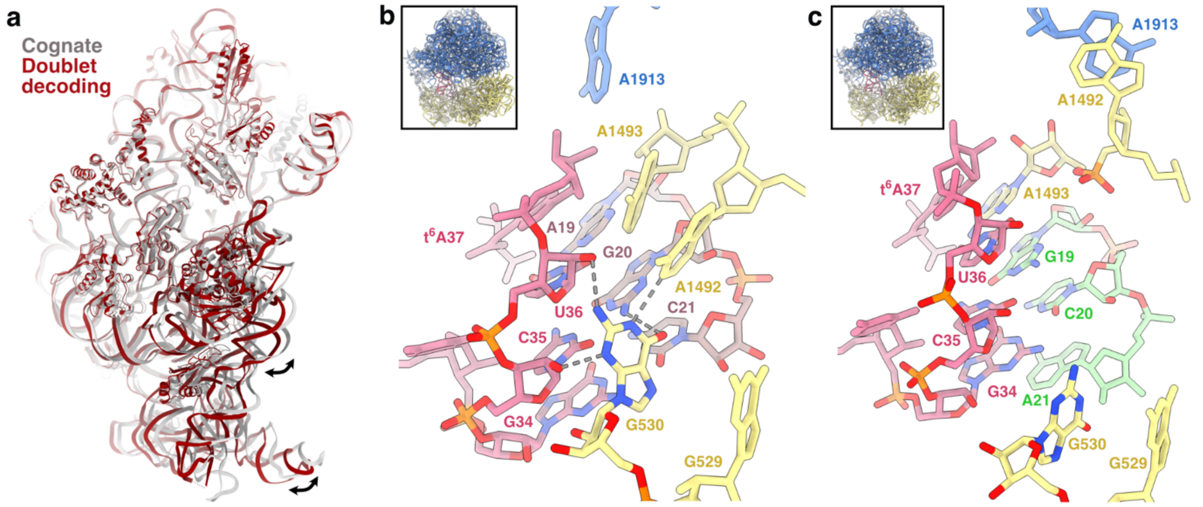

Supplementary Figure 10. Comparison of the 30S conformation of the cognate and doublet-decoding structures. **(a)** Superposition of 30S from cognate (gray) and doublet decoding (maroon) structure aligned on the 30S head domain showing the closed and open 30S conformations. Black arrows indicate the direction of movement. **(b)** Local environment of G530 of 16S rRNA in the cognate structure with closed 30S conformation, showing hydrogen bonds to $tRNA^{Ser3}$ (pink), mRNA-AGC (brown) and 16S rRNA bases (yellow). 23S rRNA is shown in blue. **(c)** Local environment of G530 of 16S rRNA in the doublet-decoding structure with open 30S conformation. G530 is withdrawn from the decoding center. Colors as in b, mRNA-GCA in green. Insets show zoomed-out view of the respective ribosome structure in the same orientation.

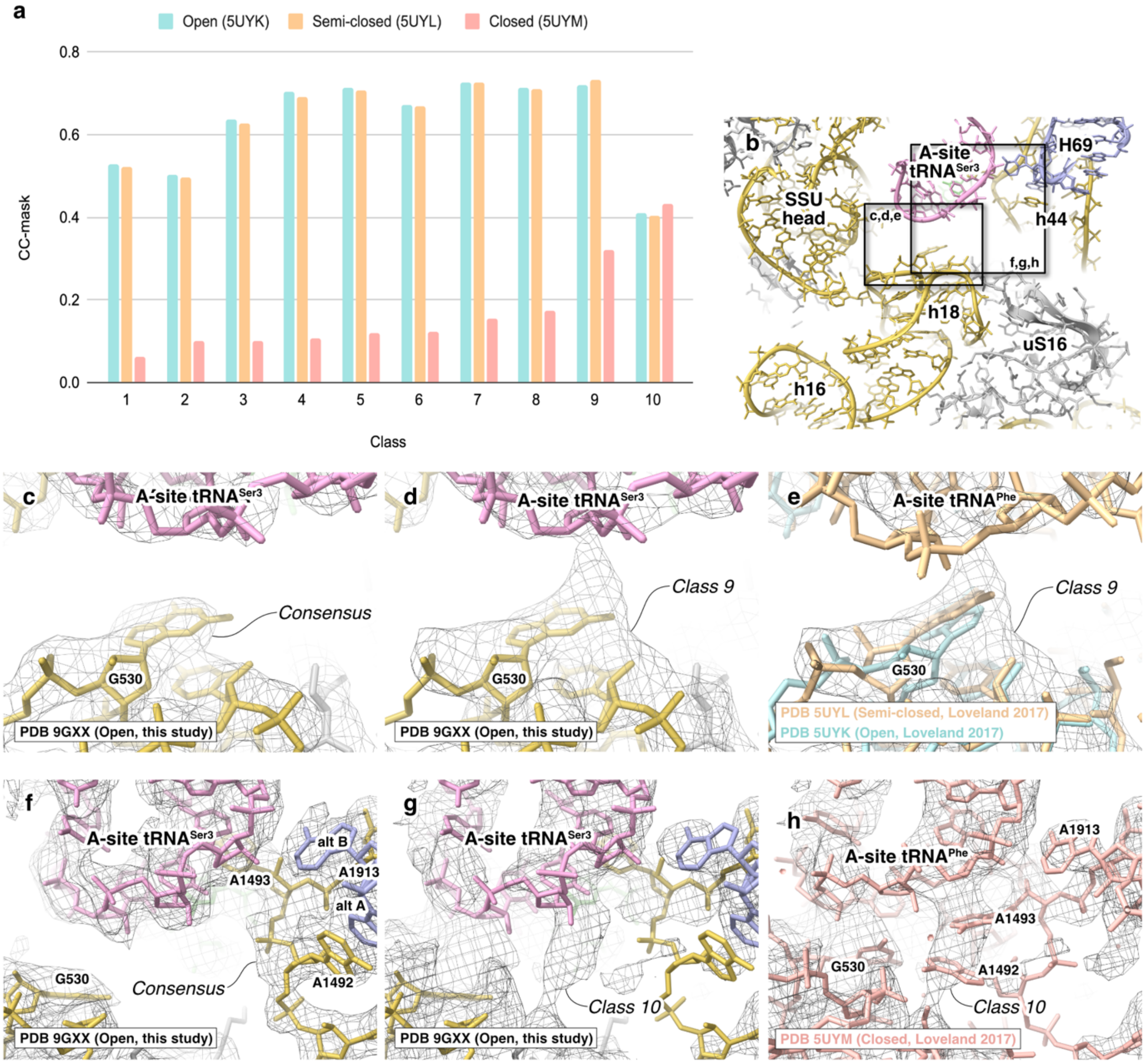

Supplementary Figure 11. Focused classification over the 30S shoulder region of the doublet-decoding structure and comparison with open (class I, PDB 5UYK), semi-closed (class II, PDB 5UYL) and closed (class III, PDB 5UYM) conformations from Loveland *et al.* 2017[8]. **(a)** Map-model cross-correlation for the 30S shoulder region after focused classification into 10 classes, with class 1-8 most similar to an open conformation[8], class 9 most similar to a semi-open conformation and class 10 most similar to a closed conformation. **(b)** Overview of the decoding center and 30S shoulder region. tRNA^{Ser3} is shown in pink, 16S rRNA in yellow, 23S rRNA in light blue and r-protein S12 in gray. **(c)** Close-up of G530 in the consensus map (mesh) of the doublet-decoding structure. Colors as in b. **(d)** Class 9 map (mesh) shown together with the model from the doublet-decoding structure. Colors as in b. **(e)** Class 9 map (mesh) shown together with models of open (PDB 5UYK, blue) and semi-closed (PDB 5UYL, orange) structures[8]. **(f)** Close-up of monitoring bases A1492 and A1493 in the consensus map (mesh) of the doublet-decoding structure. Colors as in b. **(g)** Class 10 (mesh) shown together with the model from the doublet-decoding structure. Colors as in b. **(h)** Class 10 (mesh) shown together with the model of a closed (PDB 5UYM, salmon) structure[8].

Supplementary references

1. González-López, A., Larsson, D. S. D., Koripella, R. K. & Cain, B. N. Structures of the Staphylococcus aureus ribosome inhibited by fusidic acid and fusidic acid cyclopentane. *Sci Rep* **14**, 14253 (2024).
2. Jenner, L. *et al.* Structural basis for potent inhibitory activity of the antibiotic tigecycline during protein synthesis. *Proc Natl Acad Sci U S A* **110**, 3812–3816 (2013).
3. Rundlet, E. J. *et al.* Structural basis of early translocation events on the ribosome. *Nature* **595**, 741–745 (2021).
4. Flis, J. *et al.* tRNA Translocation by the Eukaryotic 80S Ribosome and the Impact of GTP Hydrolysis. *Cell Rep* **25**, 2676-2688.e7 (2018).
5. Guyomar, C., D'Urso, G., Chat, S., Giudice, E. & Gillet, R. Structures of tmRNA and SmpB as they transit through the ribosome. *Nat Commun* **12**, (2021).
6. Hill, C. H. *et al.* Structural and molecular basis for Cardiovirus 2A protein as a viral gene expression switch. *Nat Commun* **12**, (2021).
7. Yi, S. H. *et al.* Conformational rearrangements upon start codon recognition in human 48S translation initiation complex. *Nucleic Acids Res* **50**, 5282–5298 (2022).
8. Loveland, A. B., Demo, G., Grigorieff, N. & Korostelev, A. A. Ensemble cryo-EM elucidates the mechanism of translation fidelity. *Nature* **546**, 113–117 (2017).
